# Disturbance of Antioxidant Enzymes and Purine Metabolism in the Ejaculate of Men Living in Disadvantaged Areas of Kyzylorda Region

**DOI:** 10.3889/oamjms.2015.085

**Published:** 2015-07-10

**Authors:** Valentihna N. Kislitskaya, Zhandos D. Kenzhin, Berikbay Zh. Kultanov, Raushan S. Dosmagambetova, Anar A. Turmuhambetova

**Affiliations:** *Karaganda State Medical University, Department of Obstetrics and Gynecology, Molecular Biology and Medical Genetics, Gogol 40, Karaganda 1000008, Kazakhstan*

**Keywords:** Environmental disaster, pesticides, heavy metals, catalase, adenosine deaminase, antioxidant status, oxidative stress

## Abstract

**AIM:**

Objective of the study was to evaluate the state of the main indicators of antioxidant status and enzymes of purine metabolism in the germ cells of men living in the zone of ecological catastrophe Aral Sea region.

**METHODS:**

The criterion for inclusion is the stay of an adult in the Aral Sea area is not less than 5 years, employment in occupations with no more than 2 hazard class. Determination of the activity of adenosine deaminase (ADA) was conducted in semen by the method of Nemechek et al., 1993. Determination of the activity of catalase (CAT) was performed according by the method of Korolyuk et al., 1988.

**RESULTS:**

Results of the study indicate a change in the activity of catalase and adenosine deaminase, due to increased levels of oxidative stress and the development of the pathological process.

**CONCLUSIONS:**

According to the results of study, it was put the influence of negative factors of the Aral Sea region in men’s sperm of reproductive age gives to disability free-radical processes, that proves changing of ferments of ant oxidative protection Catalase and adenosine deaminase (ADA). This disturbance in men’s sperm of reproductive age leading to increased level of oxidative stress and impaired activity of antioxidant enzymes and purine metabolism, responsible for the abnormal transmembrane and intracellular processes, reflecting the degree of imbalance of enzymes.

## Introduction

The Aral Sea crisis is recognized as one of the global environmental problems of our time. Extreme ecological situation in the Aral crisis caused a massive chemical pollution of the territory for decades with high doses of pesticides, herbicides; discharge of industrial wastes into the rivers that feed the Aral Sea [1, 2].

Modern negative trends in the state of the environment and changes in health outcomes to date acquired special significance for Kazakhstan. To a large extent, these problems relate to the residents of Kyzylorda region, which are influenced by a complex of specific risk factors due to the consequences of environmental degradation due to environmental tragedy of the Aral Sea [3].

A large public health problem is the impact of pesticides on human health. Extremely poor conditions of storage and uncontrolled use continue to pollute the environment of the region. Therefore, organochloride pesticides found in high concentrations in soils and groundwater in the water of the Syr Darya and blood surveyed residents.

In recent years, the world in general and in particular in the Aral Sea region, much attention is paid to influence on the human body and reproductive function of heavy metals, especially lead. Lead enters the environment with exhaust gases of vehicles, used as fuel leaded gasoline emissions processing enterprises, with drainage water and dust from the dried bed of the Aral Sea [4, 5].

Environmental degradation impact on the health status of the population. Numerous studies conducted by scientists of Kazakhstan, it is shown that the health status of the population in recent decades, the Aral Sea region continues to deteriorate [6-8].

In recent years, the government is making significant steps to address socio-economic problems that have emerged in the Kyzyl-Orda region. Their implementation has contributed to some improvement in the health of a region. However, the scale of the environmental disaster in the Aral Sea region requires a pressing continuation of work to resolve them [9].

With pressure from the techno genic pollution leading role played by factors of antioxidant protection, including catalase activity. Most adverse environmental conditions cause disturbances in coordination immune-metabolic processes that lead to lower balance of adaptive reactions of the organism, one of which serves as a balance of lipid peroxidation and antioxidant activity, as well as the activity of enzymes of purine metabolism, since it is known that the activity of adenosine deaminase (ADA), a key enzyme of the purine Metabolism are closely related immunological processes in the body. On the activity of the enzyme depends on the intracellular concentration of adenosine and deoxy adenosine purine metabolites that play an important role in the cooperative interactions of immune system cells. Violation of ADA activity and following this many changes resulting from accumulation of toxic concentrations in the cell adenosine and deoxy adenosine nucleotides appropriate, may stop the biosynthesis of the RNA, DNA, and cell death [10].

All of the above identified urgency of the problem and has served as the theoretical basis for the clinical and laboratory research.

The aim of the study was to evaluate the state of the main indicators of antioxidant status and enzymes of purine metabolism in the germ cells of men living in the zone of ecological catastrophe Aral Sea region.

## Material and Methods

The ethical issues have been provided in presented research with the percussive agree of studied patients. Clinical and laboratory studies were conducted at 2 population settlements (Aralsk, Ayteke Bi, Kyzyl-Orda region). Healthy people without diseases of urogenital system and sexually transmitted diseases were studied in this research. Subjects were divided into the following age groups: 18-19 years, 20-29 years, 30 to 39 years, and 40 - 49 years. The criterion for inclusion is the stay of an adult in the Aral Sea area is not less than 5 years, employment in occupations with no more than 2 hazard class.

Determination of the activity of adenosine deaminase (ADA) was conducted in semen method Nemechek et al., [11]. ADA activity was evaluated by the rate of decrease in adenosine sodium phosphate buffer. For the study was prepared by potassium-phosphate buffer which is a mixture of 150 ml of 0.2M KH_2_PO_4_ and 117.3 ml of 0.2 M KOH solution. The mixture was adjusted to 600 ml with distilled water with the addition of 6.6 mg of adenosine, in pH 7.4 buffer. Next, a mixture of 3 ml of buffer and 0.2 ml of the lysate was centrifuged for 5 min at 3000 rev/min. Determination of adenosine deaminase was carried out in a spectrophotometer with respect to potassium-phosphate buffer at a wavelength of 265 nm.

Determination of the activity of catalase (CAT) was performed according to the method Korolyuk et al., [12]. The method is based on the ability to form hydrogen peroxide with salts of molybdenum stable colored complex. Preparation of the solution of hydrogen peroxide to the 100 ml of distilled water was added 0.1 ml of 33% H_2_O_2_.

Statistical analysis was performed using the package STATISTICA 6.0 (Stat- Soft, 2001) and the program BIOSTATISTICA 4.03 [20]. The data in the text and tables are presented as M (25, 75), where Me -median; 1st and 3rd quartiles. For the sample means was indicated 95% confidence interval. The critical level of significance when testing statistical hypotheses set at 0.05 (p <0.05).

## Results

It can be seen from the Table 1 that the enzymes of purine metabolism are changing. Adenosine desaminase (ADA) in sperm of men with 18-20 years age, living in the Area of Aral Sea region, was lower in comparison with the age group 30-39 years (17% decrease). In comparison with the age group 40-49 years, the activity of adenosine desaminaze was higher in comparison with the age groups 18-29 and 30-39 years.

**Table 1:** Indicators and AOP enzymes of purine metabolism in the semen of men living in the disaster zone, M ± m

Regions/Indicators	The studied group	Catalase (CAT), mol H_2_O_2_ ml/min	Adenosine desaminase (ADA), adenosine nmol/ml/min
Ecological catastrophe (Aiteke-bi. Aralsk)	18-29 years	1.12 ± 0.10	5.99 ± 0.54

	30-39 years	1.02 ± 0.10	4.97 ± 0.46

	40-49 years	1.27 ± 0.09	6.19 ± 0.49

A comparison of the nature of changes in the activity of antioxidant enzymes and purine metabolism in different age groups showed a mixed trend in the activity of ADA with more pronounced suppression observed in the group of 30-39 years, while the comparison active spacecraft revealed the highest level of group 40-49 years.

## Discussion

In assessing changes in the activity of enzymes catalase, we traced the activity of enzymes. Comparison of the indicators of catalase activity in men’s sperm of 3 age groups did not reveal the sufficient abnormalities.

Elevated levels of catalase and adenosine deaminase in the age group of 40-49 men were found, and these changes are unidirectional. In our opinion this results are connected with disturbance of affecting change in the structure of cell membranes, which serve as an early warning sign of trouble homeostatic and development of the pathological process, which is consistent with a number of results obtained in a survey of men exposed to a complex of negative factors, which is consistent with previous studies [13].

It is known that under conditions of prolonged exposure to adverse environmental factors on the reproductive system, disturbed activity of antioxidant enzymes and purine metabolism are found (Kultanov [14, 15]). In the zone of ecological catastrophe Aiteke -bi and Aralsk of Kyzylorda region changes of antioxidant status in the semen of men of different age groups was found.

In the previous paper we published that perinatal losses in women in the ecological disaster zone are 24%. Every fourth woman has cases of spontaneous pregnancy termination and/or non-developing pregnancy in the anamnesis, which may be repeated many times. In this regard, we can assume that the effect of dust – salt aerosols high concentrations and high background radiation lead to an increase in the frequency of perinatal mortality among the examined women [16].

In our opinion, the existing negative factors in the area of environmental disaster the Aral Sea region activate free-radical processes in male germ cells, leading to increased levels of oxidative stress and impaired activity of antioxidant enzymes and purine metabolism, responsible for the abnormal transmembrane and intracellular processes, reflecting the degree of imbalance of enzymes.

In conclusion, multidirectional change in the activity of ADA and CAT in the semen of men living in the Aral Sea region might be as a result of structural damaged sperm, which leads to disruption of morphological differentiation and movement of sperm. These processes provide the energy derived from the processes of oxidative metabolism enzymes, and purine metabolism.
